# Colistin versus meropenem in the empirical treatment of ventilator-associated pneumonia (Magic Bullet study): an investigator-driven, open-label, randomized, noninferiority controlled trial

**DOI:** 10.1186/s13054-019-2627-y

**Published:** 2019-11-28

**Authors:** José M. Cisneros, Clara María Rosso-Fernández, Cristina Roca-Oporto, Gennaro De Pascale, Silvia Jiménez-Jorge, Esteban Fernández-Hinojosa, Dimitrios K. Matthaiou, Paula Ramírez, Ramón Ortiz Díaz-Miguel, Angel Estella, Massimo Antonelli, George Dimopoulos, José Garnacho-Montero, on behalf of the Magic Bullet Working Group WP1, V. Di Gravio, G. Bello, V. González, R. Leal, A. Puppo, J. A. Lepe, A. Gutiérrez-Pizarraya, E. Villarreal, M. C. Espinosa, M. J. Polanco, M. D. López, M. E. Herrera-Gutiérrez, G. Seller, S. Zakinthinos, S. Malachias, A. Komnos, T. Zafeiridis, R. Sierra, A. González, E. Zakynthinos, L. Alvárez-Rocha, M. Victoria de la Torre, P. J. Domínguez, M. Bitsan, A. Koutsoukou, R. Zaragoza, E. De Roberts, J. M. Allegue, J. Carlos Pozo, G. Nakos, R. Ferrer, I. Pneumatikos, P. Vidal, José M. Cisneros, Clara María Rosso-Fernández, Cristina Roca-Oporto, Gennaro De Pascale, Silvia Jiménez-Jorge, Esteban Fernández-Hinojosa, Dimitrios K. Matthaiou, Paula Ramírez, Ramón Ortiz Díaz-Miguel, Angel Estella, Massimo Antonelli, George Dimopoulos, José Garnacho-Montero

**Affiliations:** 1 Department of Infectious Diseases, Microbiology and Preventive Medicine, Institute of Biomedicine of Seville (IBIS), University Hospital Virgen del Rocio CSIC, University of Seville, Avenida Manuel Siurot s/n, 41013 Seville, Spain; 2 Clinical Trial Unit, Hospital Universitario Virgen del Rocío, Avenida Manuel Siurot s/n, 41013 Seville, Spain; 3 Fondazione Policlinico Universitario A. Gemelli IRCCS, Rome, Italy; 4 Intensive Care Clinical Unit, Hospital Universitario Virgen del Rocío, Avenida Manuel Siurot s/n, 41013 Seville, Spain; 5 Department of Critical Care, University Hospital ATTIKON, National and Kapodistrian University of Athens, Athens, Greece; 6 Intensive Medicine Unit, Hospital Universitario y Politécnico La Fe, Avinguda de Fernando Abril Martorell, 106, 46026 Valencia, Spain; 7 Intensive Medicine Unit, Hospital General Universitario de Ciudad Real, Calle Obispo Rafael Torija s/n, 13005 Ciudad Real, Spain; 8 Intensive Care Unit, Hospital Universitario de Jerez, Carretera Nacional IV s/n, 11407 Jerez de la Frontera, Cádiz Spain; 9 Università Cattolica del Sacro Cuore, Rome, Italy; 10 Intensive Care Clinical Unit, Hospital Universitario Virgen Macarena, Calle Dr. Fedriani, 3, 41009 Seville, Spain

**Keywords:** Colistin, Ventilator-associated pneumonia, Multidrug-resistant bacteria, Carbapenem-resistant gram-negative bacilli

## Abstract

**Background:**

Colistin is recommended in the empirical treatment of ventilator-associated pneumonia (VAP) with a high prevalence of carbapenem-resistant gram-negative bacilli (CR-GNB). However, the efficacy and safety of colistin are not well defined.

**Methods:**

A multicenter prospective randomized trial conducted in 32 European centers compared the efficacy and safety of colistin (4.5 million unit loading dose followed by a maintenance dose of 3 million units every 8 h) versus meropenem (2 g every 8 h), both in combination with levofloxacin (500 mg every 12 h) for 7–14 days in patients with late VAP. Between May 2012 and October 2015, 232 patients were randomly assigned to the 2 treatment groups. The primary endpoint was mortality at 28 days after randomization in the microbiologically modified intention-to-treat (mMITT) population. Secondary outcomes included clinical and microbiological cure, renal function at the end of the treatment, and serious adverse events. The study was interrupted after the interim analysis due to excessive nephrotoxicity in the colistin group; therefore, the sample size was not achieved.

**Results:**

A total of 157 (67.7%) patients were included in the mMITT population, 36 of whom (22.9%) had VAP caused by CR-GNB. In the mMITT population, no significant difference in mortality between the colistin group (19/82, 23.2%) and the meropenem group (19/75, 25.3%) was observed, with a risk difference of − 2.16 (− 15.59 to 11.26, *p* = 0.377); the noninferiority of colistin was not demonstrated due to early termination and limited number of patients infected by carbapenem-resistant pathogens. Colistin plus levofloxacin increased the incidence of renal failure (40/120, 33.3%, versus 21/112, 18.8%; *p* = 0.012) and renal replacement therapy (11/120, 9.1%, versus 2/112, 1.8%; *p* = 0.015).

**Conclusions:**

This study did not demonstrate the noninferiority of colistin compared with meropenem, both combined with levofloxacin, in terms of efficacy in the empirical treatment of late VAP but demonstrated the greater nephrotoxicity of colistin. These findings do not support the empirical use of colistin for the treatment of late VAP due to early termination.

**Trial registration:**

ClinicalTrials.gov, NCT01292031. Registered 9 February 2011.

## Background

Ventilator-associated pneumonia (VAP) is considered one of the most common hospital-acquired infections and the leading infectious cause of mortality in intensive care units (ICUs) [1]. Multidrug-resistant gram-negative bacilli (MDR-GNB), including *Pseudomonas aeruginosa*, *Acinetobacter baumannii*, and extended-spectrum beta-lactamase (ESBL)-producing or carbapenemase-producing *Enterobacteriaceae*, are currently the pathogens most frequently isolated from patients with late VAP in some countries [2, 3].

VAP caused by MDR-GNB is associated with high mortality, and appropriate empirical therapy is crucial for survival [4–6]. In addition, inappropriate antimicrobial treatment is administered more frequently to patients with VAP caused by these pathogens [7].

Thus, the management of patients highly suspected of having VAP caused by MDR-GNB remains an unresolved challenge. Carbapenems have been the backbone for the treatment of these infections, but the development of strains resistant to these antimicrobials raises concerns regarding their use. Currently, colistin is the antimicrobial with the greatest in vitro activity against carbapenem-resistant (CR)-GNB, and recent guidelines from Infectious Disease Society of America [8] and European Respiratory Society [9] recommend colistin combined with other antipseudomonal agents as a therapeutic option for the empirical treatment of VAP in units with high rates of resistance to antimicrobial agents from other classes, especially if *Acinetobacter* is the likely pathogen. Both expert panels recognize that no previous randomized clinical trial assessing colistin as empirical therapy for VAP has been published, which is a major limitation.

Clinical experience with colistin has resulted in contradictory findings. In some studies, the clinical efficacy of colistin was similar to that of beta-lactam antibiotics [10, 11]. Conversely, in others reports, colistin was found to be less effective and more toxic [12].

Three meta-analyses assessed the efficacy and safety of colistin for the treatment of VAP caused by MDR-GNB and concluded that colistin appears to be as effective and safe as beta-lactam antibiotics [13–15]. However, these meta-analyses have important limitations: the quality of the included studies is low, including observational studies; the results from studies with inhaled colistin are combined with those for intravenous colistin; and the drug combinations are highly variable.

Finally, three clinical trials have been performed to evaluate the use of intravenous colistin. One trial compared ampicillin/sulbactam and colistin for the treatment of MDR *A. baumannii* VAP but had a very small sample size of 28 patients [16]. Another study compared colistin monotherapy and colistin combined with rifampin in 210 patients with serious infections caused by extensively drug-resistant *A. baumannii* [17]. The third study compared colistin alone versus colistin plus meropenem for the targeted treatment of severe infections caused by CR-GNB in 406 patients, 207 of whom had VAP [18]. However, no clinical trial has been performed to evaluate the efficacy and safety of intravenous colistin for the empirical treatment of serious infections, including VAP.

We aimed to compare the efficacy and safety of empirical treatment with intravenous colistin versus meropenem in patients with late VAP in settings with a high prevalence of CR-GNB using a randomized trial design.

## Methods

### Study design and patients

Magic Bullet is an investigator-driven, open-label, randomized controlled noninferiority trial conducted from May 2012 to November 2015. Thirty-two hospitals participated in the study—17 in Spain, 10 in Greece, and 5 in Italy. The study protocol has been published previously [19].

Patients were eligible if they were 18 years of age or older, developed VAP after at least 96 h on mechanical ventilation or less than 96 h on mechanical ventilation if they had previously received antibiotic treatment for at least 5 days, and were hospitalized for more than 7 days. VAP was defined as new or progressive pulmonary infiltrates on a chest X-ray suggestive of pneumonia with no other probable cause in combination with at least one of the following characteristics: fever, an elevated (> 10,000/mm) total peripheral white blood cell (WBC) count, immature neutrophils (bands) higher than 15% regardless of the total peripheral WBC count, leukopenia with a total WBC count less than 4500/mm, new onset of expectorated or suctioned respiratory secretions characterized by a purulent appearance indicative of bacterial pneumonia, or a Modified Clinical Pulmonary Infection Score (CPIS) > 4.

Specific exclusion criteria included the isolation of colistin- or meropenem-resistant GNB cultures from respiratory samples from surveillance cultures in the 7 days before inclusion, as well as the use of meropenem. The complete inclusion and exclusion criteria are detailed in Additional file 1.

The study was conducted in accordance with the Declaration of Helsinki and with the legal norm directive 2001/20/EC of the European Parliament relating to the implementation of Good Clinical Practice. The institutional ethics committee at each study site approved the protocol, and the regulatory authorities of the three countries provided authorization. All patients or their legal surrogates provided written informed consent prior to study enrollment.

The study was monitored for safety by the Data Safety Monitoring Board (DSMB) and assessed when 50% of the sample size was recruited to detect and report early evidence of a prespecified or unanticipated benefit or detriment to trial participants that may be attributable to one of the treatments under evaluation to determine recommendations concerning continuation or conclusion of the trial [19]. The trial was registered with ClinicalTrials.gov, number NCT01292031, and with EudraCT, number 2010-023310-31.

### Randomization and masking

After confirmation of the inclusion/exclusion criteria by the local principal investigator in an electronic case report form, patients were randomized to receive colistin or meropenem in a 1:1 allocation using an automatic system integrated into the electronic case report form. The overall study population included all patients who were enrolled, randomly assigned, and received at least one dose of the study medication constituted the modified intention-to-treat (MITT) population.

Randomization was stratified by patient severity at the time of VAP diagnosis measured with the Acute Physiology and Chronic Health Evaluation (APACHE) score (≤ 15 or > 15) and by clinical site. Given the open-label trial design, masking was not applicable.

### Procedures

Patients received either colistin methanesulfonate through a 1-h intravenous infusion at a loading dose of 4.5 million units (MIU), followed by 3 MIU maintenance doses every 8 h in a 30-min infusion, or meropenem at a dose of 2 g administered intravenously (i.v.) every 8 h in a 30-min infusion. In addition, all patients received levofloxacin (500 mg i.v. every 12 h in a 30-min infusion). For patients with moderate renal dysfunction at baseline, the colistin dosage was adjusted as follows: 2 MIU every 12 h with a creatinine clearance (CrCl) of 50–90 ml/min, and 2 MIU every 24 h with a CrCl of 10–50 ml/min following the formula of Gounden et al. [20]. For patients with a CrCl of 10–50 ml/min, the dose of meropenem was reduced to 1 g every 12 h, and levofloxacin was reduced to 250 mg every 12 h.

The combination with levofloxacin in both groups was based on the results of the clinical trial of Heyland et al. [21], which suggested that for patients at a high risk of MDR-GNB, meropenem and ciprofloxacin combination therapy is safe and may be associated with better microbiological and clinical outcomes.

The drugs used in this study were commercial preparations of colistin and meropenem. Colistin is manufactured by G.E.S., Genéricos Españoles, Madrid (Spain), and meropenem is manufactured by Fresenius Kabi España, Barcelona (Spain). Both drugs were purchased and labeled for the study. Antimicrobials with activity against methicillin-resistant *Staphylococcus aureus* (vancomycin or linezolid) were administered at the investigator’s discretion [19].

A respiratory sample for microbiological diagnosis was obtained from all patients before randomization. The method used (bronchoalveolar lavage (BAL) or tracheal aspirate) was freely selected by the investigator. All samples were processed quantitatively. The cutoff values used to diagnose pneumonia were > 10^6^ colony-forming units per ml (CFU/ml) for conventional tracheal aspirates and > 10^4^ CFU/ml for BAL fluid. A set of two blood cultures was also obtained to determine the presence of bacteremia. Once the microbiological results from baseline samples were available, the therapy was adapted to the culture results. As a general rule, physicians were advised to prescribe a single antibiotic with the narrowest spectrum that had activity against the infecting organism as soon as possible.

To evaluate a microbiological cure, a tracheal aspirate or a sputum culture was collected if the patient remained on mechanical ventilation or if the patient breathed spontaneously, at 72 h, at 8 days, at the end of treatment, and at 28 days after randomization. Pathogen identification and susceptibility testing for all isolates were performed by the local laboratory. To test the susceptibility to colistin in most hospitals participating in the study, commercial microdilution was used (83%), and in the remaining hospitals, gradient strips were used (17%). Colistin monitoring was not performed for dosing adjustments.

Assessments of clinical symptoms and physical findings, sample collection, and evaluations of efficacy and safety variables, including renal function monitoring (CrCl and renal replacement therapy) and adverse events, were conducted at the baseline evaluation, at 72 h, at 8 days, at the end of treatment, and at 28 days after randomization.

### Outcomes

The primary outcome of efficacy was determined according to the proportion of patients in the MITT population with microbiologically confirmed (microbiologically modified intention-to-treat, mMITT) VAP who died from any cause 28 days after randomization.

The secondary outcomes included 28-day all-cause mortality; clinical cure; renal function at the end of treatment, including post hoc renal failure based on the RIFLE score [22]; adverse events in the MITT population; and microbiological cure in the mMITT population. A post hoc analysis of mortality in the group with CR-GNB VAP was added. Clinical cure was defined as complete resolution of all signs and symptoms of VAP at 28 days after randomization. Microbiological cure was defined as eradication of the pathogen causing VAP at 28 days after randomization.

Adverse events included any serious treatment-emergent adverse events (SAEs), suspected SAEs related to a study medication (according to the investigator’s opinion), and serious unexpected adverse events up to 28 days after the last dose of the study medication.

### Statistical analysis

The noninferiority of colistin was confirmed if the lower limit of the two-sided 95% confidence interval (CI) for the difference in mortality rates (colistin minus meropenem) in the mMITT population was less than or equal to 10% [23]. On the assumption of a success rate of 20% mortality [21], a sample of 198 patients who could be evaluated in each treatment group (a total of 396) was required to demonstrate noninferiority at the two-sided significance level of 5% with a power of 80%. Considering that the microbiological diagnosis of VAP is obtained in approximately 80% of episodes [21], a 25% increase in the sample size is required. Thus, a total of 496 individuals were needed. The primary analysis was performed by intention-to-treat in the mMITT and MITT populations.

Continuous variables were reported as the means (standard deviations (SD)) or the medians (interquartile ranges), and categorical variables were reported as numbers (%). For dichotomous efficacy outcomes, risk ratios (RRs) and risk differences for the primary and secondary endpoints with 95% CIs were calculated with Cochran’s Mantel-Haenszel method for estimation of the common treatment effect. A Kaplan–Meier curve was plotted for time to death until day 28 for the mMITT and MITT populations. A *p* value < 0.05 was considered significant.

Statistical analysis was performed using SPSS version 19.0 for Windows (SPSS Inc., Chicago, IL, USA).

## Results

Based on the results from the interim analysis with half of the sample size achieved, the DSMB suggested stopping the clinical trial considering that continuation could be a risk for the safety of patients due to the high nephrotoxicity observed in the colistin group. Following this suggestion, the trial was stopped on October 13, 2015, and this decision was communicated to the ethics committees in the three countries participating in the study. The last patient visit was performed on November 3, 2015.

**Fig. 1:**
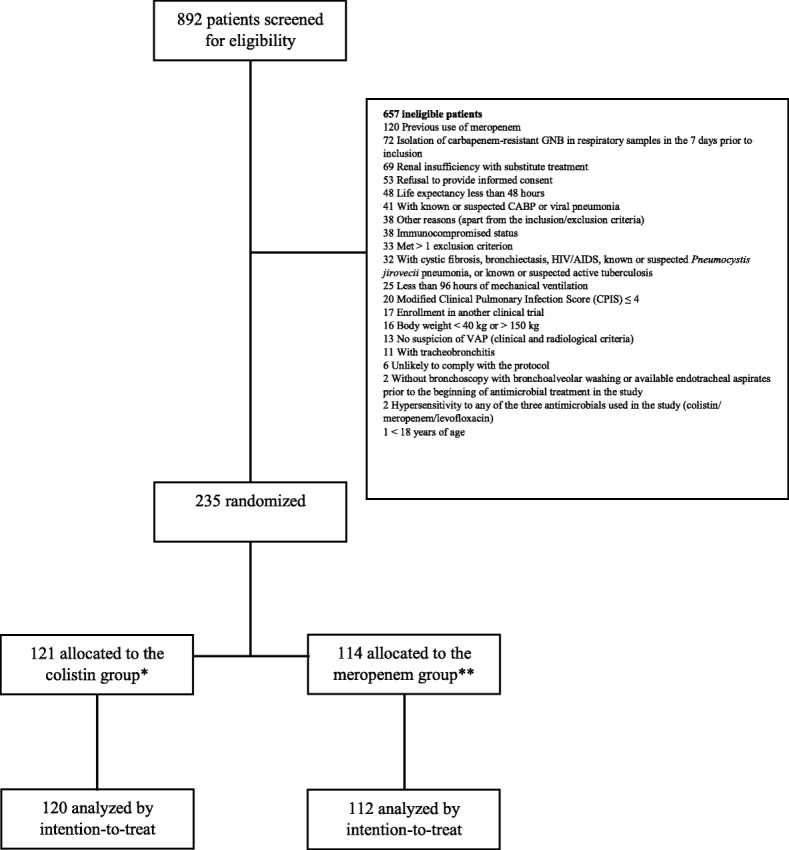
Flow chart. *One patient was excluded from the analysis due to incorrect inclusion (renal replacement therapy). **Two patients were excluded from the analysis due to incorrect inclusion (renal replacement therapy and previous use of meropenem). GNB: gram-negative bacilli; CABP: community-acquired bacterial pneumonia; HIV/AIDS: human immunodeficiency virus infection and acquired immune deficiency syndrome; CPIS: Clinical Pulmonary Infection Score; VAP: ventilator-associated pneumonia

Of the 892 patients screened for eligibility from May 2012 to October 2015, 657 were excluded, mainly due to previous use of meropenem (120/657, 18.3%). Detailed reasons for exclusion are depicted in Fig. 1. A total of 235 patients were randomized. Three patients were excluded after randomization but before receiving the study medication: two were excluded for receiving renal replacement therapy at randomization, and one was excluded for having received meropenem therapy prior to inclusion. Finally, 232 patients were analyzed for intention-to-treat in the MITT population; 120 (51.7%) were randomly allocated to the colistin plus levofloxacin group (colistin group), and 112 (48.3%) were randomly allocated to the meropenem plus levofloxacin group (meropenem group) (Fig. 1).

At the time of VAP diagnosis, the baseline characteristics of the patients (age, sex, underlying diseases, clinical presentation, APACHE score, radiographic film, CPIS, renal function, bacteremia, and microbiological diagnosis) were similar in both groups (Table 1).

**Table 1:** Patient, infection, and treatment characteristics

	Colistin + levofloxacin group (*n* = 120)	Meropenem + levofloxacin group (*n* = 112)	*p* value
Age, years (*n* = 232)	63 [49–71]	60.5 [50–70]	0.370
Female (*n* = 232)	28 (23.3)	31 (27.7)	0.448
Underlying diseases (*n* = 232)	41 (34.2)	38 (33.9)	0.969
Diabetes mellitus	19 (15.8)	22 (19.6)	0.447
Chronic liver disease	6 (5.0)	5 (4.5)	0.848
Congestive heart failure	2 (1.7)	2 (1.8)	0.945
Chronic renal disease	1 (0.8)	4 (3.6)	0.151
Chronic obstructive pulmonary disease	21 (17.5)	10 (8.9)	0.055
Neoplasia	5 (4.2)	3 (2.7)	0.535
Infection characteristics (*n* = 232)			
Days from MV to VAP	8 [6–11]	7.5 [6–11]	0.844
Clinical presentation			
Sepsis	67 (55.8)	62 (55.4)	0.992
Severe sepsis	27 (22.5)	26 (23.2)	
Septic shock	26 (21.7)	24 (21.4)	
APACHE II trial inclusion—VAP diagnosis	19 [14–24]	17 [13–22]	0.065
APACHE II ≤ 15	40 (33.3)	46 (41.1)	0.223
APACHE II > 15	80 (66.7)	66 (58.9)	
Multilobar infiltrates (Rx film)	69 (57.5)	54 (48.2)	0.157
Baseline CPIS	6 [4–7]	5 [4–7]	0.662
Baseline creatinine clearance (mg/h) (*n* = 231)	101.0 [70.9–131.0]	113.0 [66.8–163.4]	0.424
Microbiological diagnosis (*n* = 157)	82 (52.2)	75 (47.8)	0.824
VAP caused by *A. baumannii*, *P. aeruginosa*, and *K. pneumoniae* (*n* = 79)	40 (48.8)	39 (52)	0.811
MIC distribution			
Meropenem MIC			
≤ 2 mg/l—susceptible	16 (40)	18 (46.2)	0.719
> 2–8 mg/l—intermediate	4 (10)	5 (12.8)	
> 8 mg/l—resistant^1^	20 (50)	16 (41)	
Colistin MIC			
≤ 2 mg/l—susceptible^2^	35 (87.5)	32 (82.1)	0.500
> 2 mg/l—resistant	5 (12.5)	7 (17.9)	
Levofloxacin MIC			
≤ 2 mg/l—susceptible	16 (40)	17 (43.6)	0.746
> 2 mg/l—resistant	24 (60)	22 (56.4)	
Bacteremia (n = 232)	21 (17.5)	17 (15.2)	0.633
Treatment (*n* = 232)			
Empirical treatment with vancomycin	19 (15.8)	19 (17)	0.816
Empirical treatment with linezolid	17 (14.8)	18 (16.4)	0.744
Appropriate empirical antibiotic treatment (*n* = 157)	65 (79.3)	54 (72)	0.288
Duration of antibiotic treatment			
Days of treatment (*n* = 232)	9 [5–14]	7.9 [5–11]	0.035
Days of levofloxacin (*n* = 232)	8 [5–13]	7 [4–9]	0.003

The data are expressed as *n*, *n* (%), and the median (IQR) unless otherwise indicated

*MV* mechanical ventilation, *VAP* ventilator-associated pneumonia, *APACHE* Acute Physiology and Chronic Health Evaluation, *Rx* radiographic film, *GNB* gram-negative bacilli, *CPIS* Clinical Pulmonary Infection Score

^1^Definition according to the EUCAST 2012 recommendations and criteria. Carbapenem-resistant gram-negative bacteria (MIC > 8 mg/l). Colistin-resistant gram-negative bacteria (MIC > 2 mg/l for *A. baumannii* and *Enterobacteriaceae* and > 4 mg/l for *P. aeruginosa*). EUCAST. Breakpoint tables for interpretation of MICs and zone diameters version 2.0, valid from 2012 to 01-01. http://www.eucast.org/ast_of_bacteria/previous_versions_of_documents/

^2^Includes *P. aeruginosa*; all of these isolates had an MIC breakpoint ≤ 4 mg/l (susceptible)

VAP was microbiologically confirmed in 157/232 (67.7%) patients, who represent the mMITT population; 82 (52.2%) patients were included in the colistin group, 75 (47.8%) patients were included in the meropenem group, and 36 patients (22.9%) had VAP caused by CR-GNB. In the respiratory samples, the microbiological diagnosis of VAP was confirmed in 141/208 (68%) of the tracheal aspirates and in 16/24 (67%) of the BAL samples. Bacteremia was detected in 38/232 (16.4%) cases of VAP, and the etiology of VAP was polymicrobial in 55/157 (35.0%) patients (Table 2).

**Table 2:** Overall etiology of ventilator-associated pneumonia and the distributions by group

Pathogens	Number (%)	Colistin + levofloxacin group (%)	Meropenem + levofloxacin group (%)	Risk ratio (95% CI)	*p* value
Total	212	108	104		
Gram-negative bacilli	174 (82.1)	89 (82.4)	85 (81.7)		
*Acinetobacter baumannii*	34 (16.0)	16 (14.8)	18 (17.3)	0.83 (0.39, 1.74)	0.621
*Pseudomonas aeruginosa*	34 (16.0)	19 (17.6)	15 (14.4)	1.27 (0.60, 2.69)	0.530
*Klebsiella pneumoniae*	29 (13.6)	14 (13)	15 (14.4)	0.88 (0.40, 1.96)	0.757
*Escherichia coli*	20 (9.4)	12 (11.1)	8 (7.7)	1.50 (0.58, 4.01)	0.395
*Enterobacter aerogenes*	13 (6.1)	6 (5.6)	7 (6.7)	0.82 (0.25, 2.60)	0.721
*Enterobacter cloacae*	10 (4.7)	5 (4.6)	5 (4.8)	0.96 (0.25, 3.68)	0.951
*Proteus mirabilis*	7 (3.3)	2 (1.9)	5 (4.8)	2.76 (0.52, 14.51)	0.213
*Klebsiella oxytoca*	6 (2.8)	4 (3.7)	2 (1.9)	0.53 (0.10, 2.94)	0.458
*Serratia* spp.	5 (2.3)	4 (3.7)	1 (0.9)	0.26 (0.03, 2.37)	0.201
*Haemophilus influenzae*	4 (1.8)	1 (0.9)	3 (2.9)	3.28 (0.34, 31.96)	0.281
*Citrobacter freundii*	4 (1.8)	1 (0.9)	3 (2.9)		
*Citrobacter koseri*	3 (1.4)	2 (1.9)	1 (0.9)	0.53 (0.05, 5.94)	0.602
*Stenotrophomonas maltophilia*	3 (1.4)	2 (1.9)	1 (0.9)	0.53 (0.05, 5.94)	0.602
*Achromobacter xylosoxidans*	2 (1.0)	1 (0.9)	1 (0.9)	1.07 (0.07, 17.35)	0.961
Gram-positive aerobes	38 (17.9)	19 (17.6)	19 (18.3)		
*Staphylococcus aureus*	26 (12.2)	14 (13)	12 (11.5)	1.14 (0.50, 2.66)	0.751
*Enterococcus* spp.	8 (3.8)	3 (2.8)	5 (4.8)	1.82 (0.43, 7.81)	0.413
*Streptococcus pneumoniae*	3 (1.4)	1 (0.9)	2 (1.9)	2.16 (0.19, 24.20)	0.521
*Streptococcus* sp.	1 (0.004)	1 (0.9)	0 (0.0)	–	0.325
Total	212 (100)^a^	108 (100)	104 (100)		

^a^55 cases of VAP were polymicrobial. *CI* confidence interval

In total, 212 bacteria were isolated, mainly GNB (174/212, 82.0%). The most frequent GNB were *P. aeruginosa*, *A. baumannii*, and *Klebsiella pneumoniae* (97/212, 45.8%). Of them, 37/97 (38.1%) were CR, and 12/97 (12.4%) were colistin resistant. All pathogens isolated at study entry are presented in Table 2.

Optional empirical treatment with linezolid or vancomycin and appropriate empirical treatment received 31.4% (73/232) and 75.8% (119/157), respectively. The duration of therapy was higher in the colistin group (9 days (5 to 14)) than in the meropenem group (7.9 days (5 to 11), *p* = 0.035) (Table 1).

**Fig. 2:**
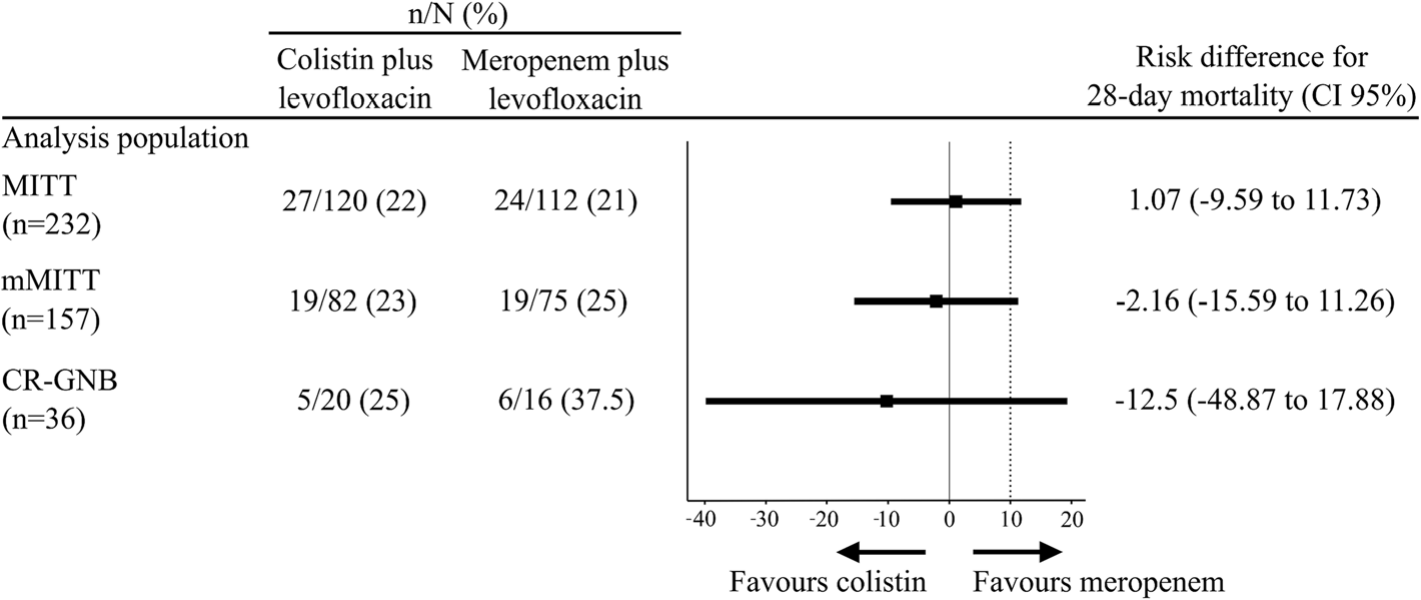
Twenty-eight-day all-cause mortality outcomes. mMITT, microbiologically modified intention-to-treat; MITT, modified intention-to-treat; CR-GNB, carbapenem-resistant gram-negative bacilli. The dotted lines represent the noninferiority margins at ≤ 10%

**Fig. 3:**
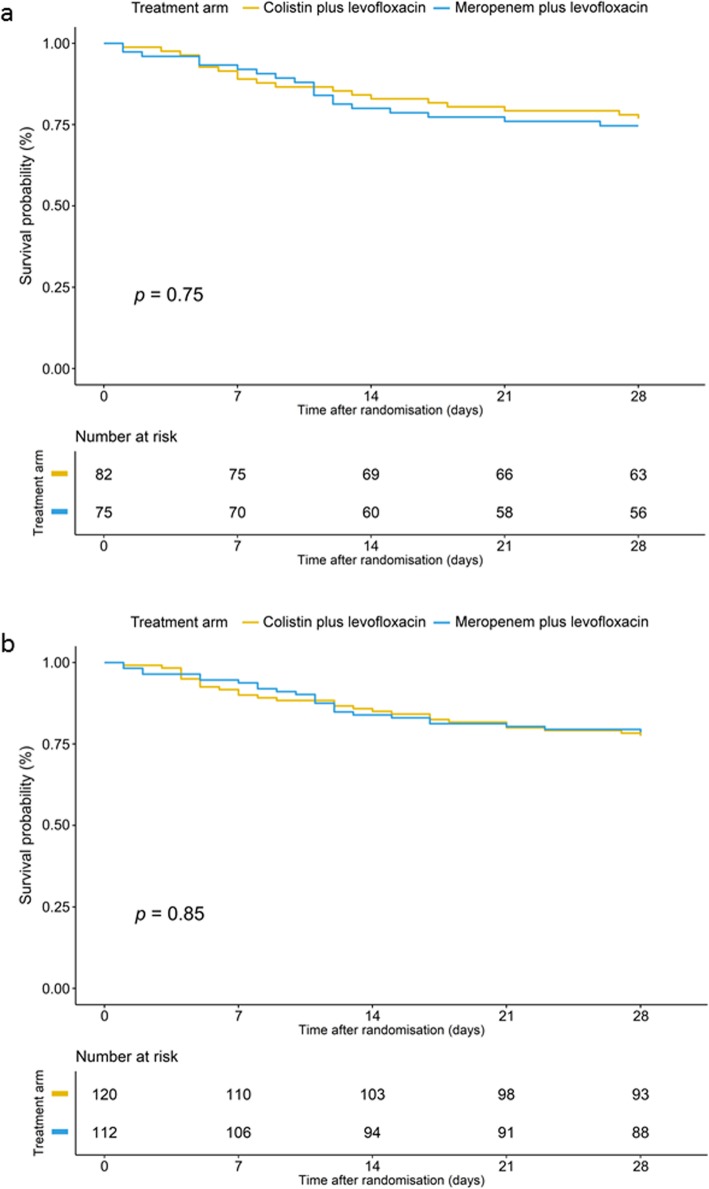
**a** Survival analysis to day 28 after randomization, the microbiologically modified intention-to-treat population. **b** Survival analysis to day 28 after randomization, the modified intention-to-treat population

For the primary outcome, all-cause mortality within 28 days after randomization in the mMITT population, no significant difference was observed between the colistin (19/82, 23%) and meropenem groups (19/75, 25%) (*p* = 0.752). The risk ratio for mortality with colistin was 0.91% (0.52 to 1.59), and the risk difference was − 2.16% (− 15.59 to 11.26), exceeding the noninferiority margin of 10% (Table 3 and Fig. 2). No survival benefit was observed in the mMITT population (log rank (Mantel-Cox) *p* = 0.75) (Fig. 3a) or in the MITT population (log rank (Mantel-Cox) *p* = 0.85) (Fig. 3b).

**Table 3:** Primary and secondary outcomes

	Colistin + levofloxacin group	Meropenem + levofloxacin group	Risk ratio (95% CI) for the outcome with colistin	*p* value
Efficacy				
Primary outcome				
Mortality at 28 days in the mMITT population (*n* = 157)	19/82 (23.2)	19/75 (25.3)	0.91 (0.53, 1.59)	0.752
Secondary outcomes				
Mortality at 28 days in the MITT population (*n* = 232)	27/120 (22.5)	24/112 (21.4)	1.05 (0.65, 1.71)	0.844
Mortality at 28 days with CR-GNB (*n* = 36)	5/20 (25.0)	6/16 (35.2)	0.67 (0.25, 1.79)	0.425
Clinical cure in the MITT population (*n* = 232)	82 (68.3)	81 (72.3)	0.94 (0.80, 1.12)	0.507
Microbiological cure in the mMITT population (*n* = 157)	46 (56.1)	42 (56.0)	1.00 (0.76, 1.32)	0.990
Microbiological failure in the mMITT population (*n* = 157)	33 (40.2)	31 (41.3)	0.97 (0.67, 1.42)	0.899
Microbiological relapse in the mMITT population (*n* = 157)	3 (3.7)	2 (2.7)	1.37 (0.24, 7.99)	0.723
Safety				
Serious adverse events (*n* = 232)	50 (41.7)	45 (40.2)	1.04 (0.76–1.41)	0.818
Serious adverse events* (*n* = 232)	4 (3.3)	1 (0.9)	3.70 (0.42, 32.90)	0.201
CrCl (ml/h) at the end of the treatment day (*n* = 231)	90.9 [57.2–141.9]	122.3 [86.1–185.7]	−37.93 (− 62.96, − 12.90)	0.003
Renal replacement therapy (*n* = 232)	11 (9.1)	2 (1.7)	5.13 (1.16, 22.65)	0.015
RIFLE score at the end of treatment compared with randomization (*n* = 232)^μ^	*n* = 120	*n* = 112	–	0.034^¥^
None	80 (66.7)	91 (81.3)	–	–
Risk	21 (17.5)	11 (9.8)	–	–
Injury	15 (12.5)	5 (4.5)	–	–
Failure	4 (3.3)	5 (4.5)	–	–

*CI* confidence interval, *mMITT* microbiologically modified intention-to-treat, *MITT* modified intention-to-treat, *CR-GNB* carbapenem-resistant gram-negative bacilli, *CrCl* creatinine clearance, *RRT* renal replacement treatment

*Serious adverse events suspected of being related to the study medications according to the investigator’s opinion (4 cases of renal insufficiency/renal impairment attributed to colistin and 1 case of compartment syndrome related to meropenem), (%) [range]

^μ^No patients with RRT > 4 weeks (equivalent to RIFLE score of “Loss”) and no patients with permanent dialysis > 3 months (equivalent to RIFLE score of “End Stage Kidney Disease”)

^¥^*p* for trend

Regarding mortality and clinical cure in the MITT population, microbiological cure in the mMITT population, mortality among patients infected with CR-GNB, and mortality depending on MIC of meropenem/colistin, no significant differences between groups were observed (Fig. 2, Tables 3 and 4).

**Table 4:** Twenty-eight-day mortality per pathogen minimum inhibitory concentration (MIC) in the 79 episodes of *A. baumannii*, *P. aeruginosa*, or *K. pneumoniae* isolated in respiratory samples obtained at baseline

	Colistin + levofloxacin group (*n* = 120), death/total number of cases (%)	Meropenem + levofloxacin group (*n* = 112), death/total number of cases (%)	*p* value
VAP caused by *A. baumannii*, *P. aeruginosa*, and *K. pneumoniae* (*n* = 79)	40 (48.8)	39 (52)	0.811
MIC distribution			
Meropenem MIC			
≤ 2 mg/l—susceptible	4/16 (25)	5/18 (27.8)	0.885
> 2–8 mg/l—intermediate	0/4 (0)	0/5 (0)	–
> 8 mg/l—resistant	5/20 (25)	6/16 (37.5)	0.419
Colistin MIC			
≤ 2 mg/l—susceptible	9/35 (25.7)	8/32 (25)	0.947
> 2 mg/l—resistant	0/5 (0)	3/7 (42.9)	0.091

*MIC* minimum inhibitory concentration, *VAP* ventilator-associated pneumonia

A higher incidence of renal failure at the end of treatment compared with randomization, mainly corresponding to the risk and injury categories of RIFLE (40/120, 33.3%, versus 21/112, 18.8%; *p* = 0.012), was observed in patients treated with colistin plus levofloxacin. At the end of treatment, ClCr was significantly lower in the colistin group than in the meropenem group (90.9 (57.2–141.9) versus 122.3 (86.1–185.7) ml/min, *p* = 0.003), and a significantly higher proportion of patients in the colistin group needed renal replacement therapy (11/120, 9.1%, versus 2/112, 1.8%; *p* = 0.015) (Table 3). The evolution of renal function, which was measured according to baseline ClCr versus ClCr at the end of treatment, did not change significantly in the colistin group (101.0 (70.9–131.0) versus 90.9 (57.2–141.9) ml/min, *p* = 0.724) and improved in the meropenem group (113.0 (66.8–163.1) versus 122.3 (86.1–185.7) ml/min, *p* ≤ 0.001). In the colistin arm, the incidence of any form of nephrotoxicity was not statistically different (*p* = 0.443) if the empirical regimen included vancomycin (15.8%), linezolid (35.3%), or no active antibiotic against methicillin-resistant *Staphylococcus aureus* (31%).

A total of 95 (40.9%) patients presented SAEs, and 5 (2.2%) patients showed SAEs suspected of being related to a study medication. The frequency of SAEs was similar in both groups, and no serious unexpected adverse events were noted. Detailed comparisons of the primary and secondary outcomes according to the treatment groups are shown in Table 3.

## Discussion

This clinical trial is the first to analyze the efficacy and safety of intravenous colistin in the empirical treatment of late VAP. The results do not demonstrate the noninferiority of colistin compared with meropenem, both of which were combined with levofloxacin due to early termination. However, nephrotoxicity was significantly higher with colistin. This information is crucial in the era of antimicrobial resistance, where the empirical use of colistin for VAP is increasingly common in clinical practice.

This clinical trial analyzed the efficacy and safety of colistin in the empirical treatment of VAP in settings with a high prevalence of CR-GNB. Both treatment groups were similar when comparing the baseline conditions of the patients (age, sex, and underlying diseases), clinical manifestations (mechanical ventilation time, disease severity), microbiological characteristics (etiological diagnosis, CR-GNB, and bacteremia), and treatment at the time of randomization (appropriate empirical treatment and the use of vancomycin or linezolid). These baseline characteristics are similar to the characteristics of patients included in other clinical trials investigating VAP [21, 24, 25] and confirm the current predominance of GNB (81.6%) as the etiology of this infection and the high frequency of CR-GNB (23.5%) in some countries.

The mortality rates of the patients were similar in both groups (22.5% in the colistin group versus 21.4% in the meropenem group, *p* = 0.844). Comparing the mortality rate found in this study with those reported in other studies is difficult due to the heterogeneity across studies.

Heyland et al. [21] described overall mortality rates of 18.7% and 26.8% for VAP by MDR-GNB, and Kollef et al. [24] described a mortality rate of 20%, all of which are higher than the 10% and 8% mortality rates reported in two studies on nosocomial pneumonia [25, 26].

These differences among studies indicate that the prognostic factors of VAP, which are as important as the APACHE score, as well as those of bacteremia and MDR bacteria are all different. In Chastre et al.’s study, the average APACHE score was 14.7, and the bacteremia rate was 4.5% [25]. In our study, the APACHE score was 18, and the bacteremia rate was 16%. In addition, in our study, the frequency of septic shock was 21.6%. The mortality rate of VAP caused by CR-GNB is higher than that caused by susceptible bacteria. Therefore, in our study and in Paul et al.’s trial, the mortality rates were 30.6% and 44%, respectively [18].

In this study, the rate of microbiological cure was poor at 56% in both groups, which is similar to the rate of 54% (30/56) described by Heyland et al. [21] in a subgroup of patients infected with difficult-to-treat GNB, demonstrating the difficulty associated with eradicating these bacteria from the respiratory tract.

In the present trial, the combination of colistin and levofloxacin did not meet the noninferiority criterion of less than or equal to 10% as defined in the protocol, possibly because the expected sample size was not reached, which is reflected in the broad confidence interval. In fact, the efficacy of colistin for the treatment of VAP has been questioned because of its poor penetration into the lung parenchyma [27]. The potential for colistin nephrotoxicity has been a major concern as demonstrated by the contradictory results published in recent studies. In this context, some studies have shown that the nephrotoxicity rate for colistin is similar to that for treatment with standard antibiotics [13]. In contrast, one observational study showed threefold higher nephrotoxicity for colistin than for beta-lactam antibiotics [12].

Our results show that colistin is associated with significantly higher nephrotoxicity than meropenem. At the end of treatment, ClCr was significantly lower in the colistin group than in the meropenem group (90.9 (57.2–141.9) versus 122.3 (86.1–185.7) ml/min, *p* = 0.003), more often resulted in severe acute renal failure requiring renal replacement therapy (9% versus 2%, *p* = 0.015), and had a higher incidence of renal failure at the end of treatment. In our study, we used higher doses of colistin (9 MIU daily for normal renal function), which may explain this result. Indeed, an observational study and a clinical trial comparing this high dose of colistin with low doses of 4 and 6 MIU daily, respectively, showed that nephrotoxicity is associated with a high-dose regimen of colistin [28, 29]. Nation et al. [30] suggest that the dose of colistin needs to be amended daily for every 10 units of change of the creatinine clearance to avoid nephrotoxicity. Unfortunately, this method had not been described when our trial was designed.

The strengths of our study are as follows: the multicenter design, which supports the external validity of the findings, and the analysis of efficacy as the primary outcome based on the mMITT population in which the diagnosis of VAP required bacteriological confirmation. Additionally, the etiology of VAP was mainly attributable to GNB, including CR-GNB; the colistin dosage was updated; and the interim analysis was assessed by the DSMB.

This study also has several limitations. First, the design was open label due to the need to know the empirical treatment to guide adjustments when the microbiological diagnosis was received. The evaluation of microbiological results was not centralized, and the absence of a central lab probably increased the heterogeneity of the microbiological results. We consider the risk of this bias to be lower than the risk that a delay in receiving the microbiological results would cause for the patients due to the inherent delays associated with transporting samples from 32 centers in 3 different countries. Second, susceptibility to colistin may cause undetected resistance when the microdilution method is not used, which was unknown when the study was designed [31]. The evaluation of some of these isolates in a reference laboratory reinforces the idea that colistin and carbapenem resistance *A. baumannii* is probably underreported in our study [32]. Third, as we did not perform therapeutic drug monitoring of meropenem and colistin, target levels may have not been reached, what occurs in a significant proportion of critically ill patients with sepsis [33].

Fourth, meropenem was not administered by extended infusion because when the study was designed, extended infusion of meropenem was not a quality standard [21]. This recommendation appears in 2016 with the guidelines from Infectious Disease Society of America which were published after the end of the study [8]. Fifth, the lack of data of SOFA score is another limitation that restricts the chance of quantifying organ function of the patients included in this trial lessening the comparability with other studies. Finally, the calculated sample size was not achieved because the DSMB suggested early trial termination based on the interim analysis, and the noninferiority of colistin could not be demonstrated.

## Conclusions

The results of this randomized clinical trial including a large number of critically ill patients with late VAP caused mostly by MDR-GNB demonstrate that colistin is more nephrotoxic than meropenem, both of which were combined with levofloxacin, and do not demonstrate the noninferiority of colistin in terms of efficacy due to early termination. These findings do not support the empirical use of colistin in the treatment of late VAP.

## Supplementary information

**Additional file 1.** Inclusion and exclusion criteria.

## Data Availability

The datasets generated during the current study are available from the corresponding author on reasonable request.

## Abbreviations

APACHE: Acute Physiology and Chronic Health Evaluation
BAL: Bronchoalveolar lavage (BAL)
CFU: Colony-forming units
CI: Confidence interval
CPIS: Clinical Pulmonary Infection Score
CrCl: Creatinine clearance
CR-GNB: Carbapenem-resistant gram-negative bacilli
DSMB: Data Safety Monitoring Board
ESBL: Extended-spectrum beta-lactamase
ICUs: Intensive care units
i.v.: Intravenously
MDR-GNB: Multidrug-resistant gram-negative bacilli
MITT: Modified intention-to-treat population
MIU: Million units
mMITT: Microbiologically modified intention-to-treat population
RRs: Risk ratios
SAEs: Serious treatment-emergent adverse events
SD: Standard deviations
VAP: Ventilator-associated pneumonia
WBC: White blood cell
