# Supplementary material for: Colistin versus meropenem in the empirical treatment of ventilator-associated pneumonia (Magic Bullet study): an investigator-driven, open-label, randomized, noninferiority controlled trial
Source: Crit Care. 2019 Nov 28;23:383. doi: 10.1186/s13054-019-2627-y (PMC6883535; doi:10.1186/s13054-019-2627-y)
Supplement: Supplementary file 1 — Additional file 1. Inclusion and exclusion criteria. [file 13054_2019_2627_MOESM1_ESM.doc]

**Additional file 1** Inclusion and exclusion criteria

| **INCLUSION CRITERIA** | **EXCLUSION CRITERIA** |
| --- | --- |
| Age ≥ 18 years. | Renal insufficiency with substitute treatment. |
| ≥ 96 h of mechanical ventilation. | Body weight < 40 kg or > 150 kg. |
| < 96 h of mechanical ventilation + 7 days in the hospital + 5 days of antibiotic treatment. | Refractory shock or another disease with a life expectancy less than 48 h after recruitment according to the researcher. |
| **Clinical criteria for VAP**: (at least one)   - Documented fever - An elevated total peripheral white blood cell (WBC) count (WBC count greater than 10,000/mm); greater than 15% immature neutrophils (bands) regardless of the total peripheral WBC count; or leukopenia with a total WBC count less than 4,500/mm. - New onset of expectorated or aspirated respiratory secretions characterized by a purulent appearance indicative of bacterial pneumonia. | Patients with   - Known or suspected CABP or viral pneumonia - Acute exacerbation of chronic bronchitis without evidence of pneumonia - Tracheobronchitis - Primary lung cancer or another malignancy metastatic to the lungs - Cystic fibrosis, bronchiectasis, HIV/AIDS, known or suspected *Pneumocystis jirovecii* pneumonia, or known or suspected active tuberculosis. - Immunocompromise; hematologic neoplasia, solid organ transplant or congenital or acquired diseases that cause significant immunodeficiency (examples: common variable immunodeficiency), and neutropenia < 500 PMN/mm^3^. |
| **Radiological criteria for VAP**: new or progressive pulmonary infiltrate on thorax radiography that suggests pneumonia with no other probable cause. | Isolation of colistin- or meropenem-resistant GNB bacteria in respiratory samples from surveillance cultures in the 7 days prior to inclusion. |
| Modified Clinical Pulmonary Infection Score (CPIS) > 4 |  |
| Respiratory BAL secretion sample or endotracheal aspirates obtained in the 24 h prior to the beginning of antimicrobial treatment in the study. | Previous use of meropenem: current use of meropenem at the time of diagnosis is not permitted (unless a unique dose of 1000 mg was administered for initiation of empirical treatment). |
| A negative pregnancy test in women of childbearing age. |  |
| A duly signed informed consent form. |  |

VAP: ventilator-associated pneumonia; WBC: white blood cell; CPIS: Clinical Pulmonary Infection Score; BAL: bronchoalveolar lavage; CABP: community-acquired bacterial pneumonia; HIV/AIDS: Human immunodeficiency virus infection and acquired immune deficiency syndrome; GNB: gram-negative bacilli.
